# Rare case of remission of a patient with small cell carcinoma of the ovary, hypercalcaemic type (SCCOHT) stage IV: Case report

**DOI:** 10.1016/j.ijscr.2019.11.053

**Published:** 2019-11-30

**Authors:** M.P. Mathey, J. Bouquet de Jolinière, A. Major, B. Conrad, F. Khomsi, D. Betticher, M. Devouassoux, A. Feki

**Affiliations:** a Département de chirurgie gynécologique et oncologique, Pr Dr Med A. Feki MD, PhD, HFR, Hôpital Cantonal, 6 chemin des pensionnats, Fribourg 1708, Switzerland; b Département d’Oncologie médicale, Pr Dr Med D. Betticher MHA, HFR, hôpital Cantonal, 6 chemin des pensionnats, Fribourg 1708, Switzerland; c Institut de pathologie Multi-Sites des HCL, Groupements hospitaliers Sud et nord, Pr M. Devouassoux MD, PhD, Centre de biologie et pathologie sud, Bat 3D 69495 Pierre Bénite Cedex, France; d Institut fur med, OnKologie, Inselspital, 3010 Berne, Switzerland

**Keywords:** Ovarian cancer, Hypercalcaemia, Apheresis, BRG1 protein, *SMARCA4*, chemotherapy

## Abstract

**Introduction:**

Small cell carcinoma of the ovary (SCC) is a very rare (less than 1 % of ovarien neoplasia), highly undifferentiated, aggressive malignancy affecting young women and linked to a poor prognosis. Overall survive rate is very low (about 16 %). SCCOHT has recently been shown to be associated with SMARCA4 gene mutations and shows some genetic similarities to malignant rhabdoid tumors (MRT).

**Presentation of case:**

After a reminder of the clinical, histological description of the SCCOHT and concensus about the medical management, we describe the rare case of a 22 years old patient with complete remission after diagnosis of un undifferentiated SCCOHT stage IV treated by conservative surgery and high-doses chemotherapy, 30 months after diagnosis.

**Discussion:**

Thus far, no standard therapy exists for SCCOHT. Treatment modalities are surgery, chemotherapy, radiotherapy and autologous stem cell transplant after high-dose chemotherapy. Research for new treatments includes target therapy.

**Conclusion:**

Autologous stem cell transplant after high-dose adjuvant chemotherapy seems to lead to the best survival rates. Invasiveness of the treatment depends on the stage of the disease, age of the patient and her fertility-sparing desire. An international collaboration will be needed to standardise practices due of the small number of patients.

## Highlights

• Small cell carcinoma of the ovary is a very rare, highly undifferentiated, aggressive malignancy that affects young women and linked to a poor prognosis (especially women under 40 years of age). We present a 22 years old African woman with complete remission 30 months after diagnosis despite a conservative surgery and only 3 cycles of chemotherapy.
• SCCOHT seems to be a monogenic disease with mutations in the tumour suppressor gene, a chromatin remodeler: *SMARCA4* and can involve germline or somatic lines. Mutation can also be a de novo sequence. Research for new treatments includes target therapy.
• Because of the rareness of SCCOHT (about 300 cases in the world) no standard treatment guidelines for SCCOHT are established, but combination of surgical debulking and chemotherapy based on cisplatin, are common practice.
• Autologous stem cell transplant after high-dose adjuvant chemotherapy seems to lead to the best survival rates, because it allows perform doses 2- to 10-fold higher than standard chemotherapy doses.

## 1 Introduction

Small cell carcinoma of the ovary (SCC) is a very rare, highly undifferentiated, aggressive malignancy that affects young women [1] and linked to a poor prognosis [2]. First reported in 1982 by Dickersin et al. [3] there are two subtypes of SCC, the hypercalcaemic type (SCCOHT) and the pulmonary type (SCCOPT). SCCOHT is extremely rare and represents less than 1 % of ovarian neoplasia. Until 2016, about 300 cases had been reported in literature [4,5]. Despite this rareness, it is the most frequent type of ovarian cancer affecting young women, especially women under 40 years of age, with a mean age of diagnosis at 24 years [[6], [7], [8]]. This case report focuses on the SCCOHT type, which is mostly associated with paraneoplastic hypercalcaemia (two-thirds of patients). SCCOHT is difficult to diagnose due to its histological similarity to a wide variety of tumours [9]. A group recently discovered that SCCOHT is a monogenic disease [10], with mutations in the tumour suppressor gene, a chromatin remodeler: *SMARCA4* (observed in 69 % of cases), which leads to inactivation of the Brahma-related gene-1 (BRG1) protein and dysregulation of DNA replication, transcription, and repair [11,12]. However, the exact histogenesis is still unclear. The most popular hypothesis is the epithelial origin. This tumor shows close similarities to rhabdoid tumours on pathological and molecular levels [13]. SCCOHT is usually unilateral, fast-growing and is commonly associated with vascular invasion [14]. Typical immunohistochemical profile is positive for vimentin, and sometimes for cytokeratin, membrane metallo-endopeptidase named CD10, calretinin, tumor suppressor protein p53 and Wilm Tumor protein 1 [6]. Clinical presentation, such as abdominal pain, nausea and vomiting, is due to the compression of the mass but is nonspecific. Gynaecological symptoms, such as irregular menstrual cycles and infertility have also been observed [1].

According to the International Federation of Gynaecology and Obstetrics (FIGO), the staging of this disease is similar to other types of ovarian cancer, from stage 1 (confined to the ovary) to stage 4 (distant metastasis). Symptomatic and larger tumours correlate with a better prognosis due to earlier detection [15]. The exact diagnosis and staging are determined by surgery [16]. Follow-up is performed using ultrasonography, computed tomography (CT) and magnetic resonance imaging (MRI).

To date, there are no standard treatment guidelines for SCCOHT [5], but the combination of surgical debulking and chemotherapy based on cisplatin, are common practice. The aggressiveness of the treatment depends on the stage of the disease, the age of the patient and her fertility-sparing desire. However, there is no consensus about the surgical management of a tumour confined to one ovary (FIGO stage 1), especially if a radical surgery is needed [2,17,18].

We describe the case of a 22-year-old patient in full remission 30 months after diagnosis, suffering from a stage IV SCCOHT treated with conservative surgery and high-doses of chemotherapy.

This work has been reported in line with the SCARE criteria [19].

## 2 Case report

**Fig. 1:**
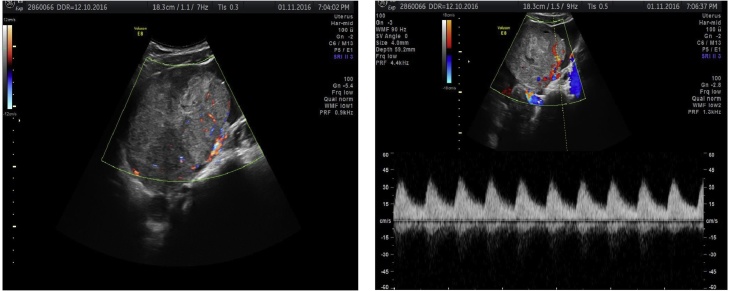
The abdominal ultrasound showes a left ovarian heterogeneous and high vascularized mass of 13 cm long axis and a moderate amount of ascites.

**Fig. 2:**
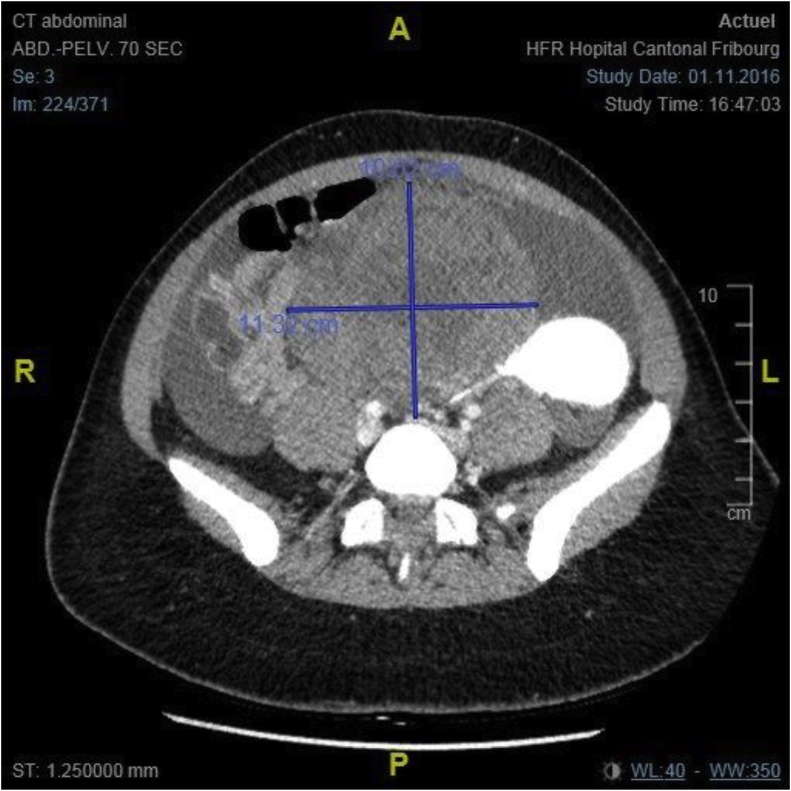
The CT Scan shows a voluminous pelvic mass of 98 × 112 × 125 mm attached to the uterus, with heterogenous enhancement and probably growing from the left ovary.

A 22-year-old nulliparous, African patient, with a history of polycystic ovary syndrome (with dysmenorrhoea and irregular periods), presented to the emergency department with diffuse abdominal pain associated with nausea, vomiting and diarrhoea. No digestive origin was found and she was addressed for a gynaecologic exam. The virginity of the patient led us to perform an abdominal ultrasound to examine the uterus and the ovaries, which showed a left ovarian heterogeneous and highly vascularised mass of 13 cm in long axis with a moderate amount of free fluid (Fig. 1 *and comments*). The uterus and the right ovary were normal. A pelvic-abdominal CT scan confirmed the gynaecologic origin of the mass (Fig. 2 *and comments*). In light of the patient’s complaint of pain and the suspicion of an ovarian torsion, we proceeded to an emergency laparoscopy for left adnexectomy and peritoneal washing. We converted to laparotomy because of the size of the tumour and its friability, and extracted to tumor without rupturing into the abdomen. Mobilisation of the uterus was difficult because of the apposition of the ovarian mass to the posterior wall of the uterus. There was no complication after surgery and prompt return of intestinal transit.

**Fig. 3:**
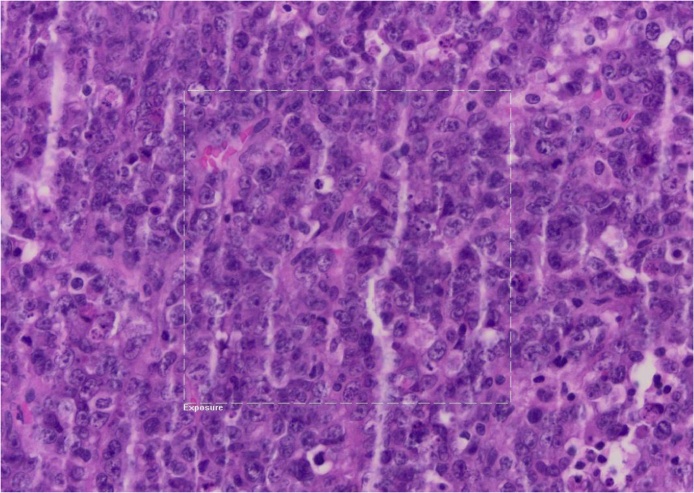
Diffused tumoral proliferation with necrotic-hemorragic composant. Small cells with hyperchromatic nucleus and irregular chromatin and very few cytoplasms and high mitosis rate. The cells are very positive with immunohistochemical markers such as Ini-1, p53, partly positive with cytokeratin AE1/AE3, WT1, CD30 and D2-40.

Classical tumour markers were elevated, with Ca-125 at 125 U/ml, Ca-19.9 at 18.4 U/l and CEA at 0.8 ng/ml, while B-HCG was negative. Histological and immunohistological features of the ovarian mass led to the diagnosis of an undifferentiated hypercalcaemic ovarian carcinoma, confirmed by two different pathologists (Fig. 3 *and comments*).

Staging was performed by positron emission tomography with CT (PET-CT) and a total-body MRI, which showed thoracic, renal, subscapular, hepatic and retroperitoneal lymphadenopathies, with a high uptake of the radioactive component. No cerebral metastasis was found. The initial staging was a stage IV, pT1c2pNxL1V1G3M1, FIGO IC2, signifying that the disease was limited to one ovary but with rupture of capsule and presence of metastasis.

Genetic mutation of *SMARCA4* was identified as a pathogenic variant in a heterogeneous state, but the screening results of her mother and father were normal.

A tumour board discussion and a second opinion from other centres in Switzerland, Paris and United Kingdom recommended performing rapid neoadjuvant chemotherapy based on cisplatin (Platino), adriamycin (Adriblastine), etoposide phosphate (Etopophos) and cyclophosphamide (Endoxan). The patient received three cycles of intensive chemotherapy less than a month after diagnosis. Side effects of the treatments such as polyneuropathy, myelotoxicity, alopecia, sterility, and dysgeusia were explained to the patient but not reported. Because of the young age and the nulliparity of the patient, a gonadotropin-releasing hormone agonist (Zoladex) was used to preserve the fertility of the patient.

The treatment plan was to provide intense neoadjuvant chemotherapy after radical surgery, and then proceed to an autologous stem cell transplantation. As planned, the patient underwent a stem cell collection after mobilisation with filgrastim (granulocyte growth factor, G-CSF) in view of bone marrow suppression because of the high-dose chemotherapy.

However, the patient refused to undergo further surgery such as a hysterectomy and right adnexectomy. She refused various treatments and accepted only the regular CT scan controls. The first thoracic and abdominal CT scans were performed three months after the end of the chemotherapy and showed no local or systemic tumour recurrence. PET-CT done five months later confirmed the absence of new hypermetabolic lesions.

Eight months after surgery, a CT scan showed a large ovarian cyst on the remaining ovary, with hypercaptation of 18 F-FDG on the PET-CT scan. Suspecting a tumour recurrence, we advised the patient to undergo surgery and new cycles of chemotherapy, but she refused. The CT scan three months later showed that the ovarian mass had spontaneously disappeared.

**Fig. 4:**
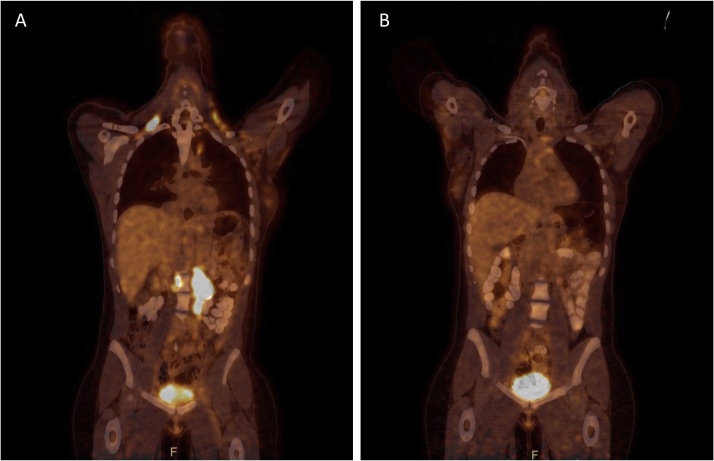
PET CT for assessment of extension showing multiple gangliotic supra and infra diaphragmatic hypercaptation (A) corresponding to metastatic adenopathy. Control PET CT in November 2018 showing the absence of tumoral recidive (B).

The last imaging control, with PET-CT, was in November 2018 and showed a total remission of the disease and at that time the patient described a general wellbeing (Fig. 4 *and comments*).

## 3 Discussion

Patient remission two years after diagnosis is a remarkable outcome, considering a SCCOHT stage IV. The stage at diagnosis is the most important prognostic factor [5], with the second factor being treatment modalities. Based on a study by Witowski et al. [5] in which a large number of patients were analysed, 41 patients survived more than five years after diagnosis, and only three died later. Given the high rate of five years recurrence, recurrence-free survival of longer than five years would be a better indicator of long-term survival. Favourable outcomes include patient age (<40 years of age), tumour size (<10 cm) and a normal pre-operative calcium level [19], which this patient had except for the tumour size which was 13 cm.

Two-thirds of patients with SCCOHT presented with pre-operative hypercalcaemia, which correlates to a parathyroid hormone related protein secreted by the tumour that binds to receptors in the bone and the kidneys [21,22]. Only a few cases reported signs and symptoms of hypercalcemia [9]. After the excision of the tumour, post-operative measures of calcium levels are usually within normal values [23]. This patient, along with 30 % of similar reported cases in the medical literature, presented no abnormality in the measured calcium level.

Thus far, no standard therapy exists for SCCOHT. Treatment modalities are surgery, chemotherapy, radiotherapy and autologous stem cell transplant after high-dose chemotherapy [24]. However, it seems that the efficiency of radiotherapy for treatment of SCCOHT is uncertain for all stages [5]. Witowski et al. evaluated the effect of treatment modalities and found that multimodal therapy of surgery, chemotherapy, and radiotherapy, which included autologous stem cell transplantation, showed the best long-term survival [11,[25], [26], [27], [28]]. Such multimodal approach has been explored for the past three decades for a variety of solid tumours in adults, especially for ovarian cancer, breast cancer and non-seminomatous germ-cell tumours and allows the administration of high-dose chemotherapy, with doses 2- to 10-fold higher than standard chemotherapy doses [29].

**Fig. 5:**
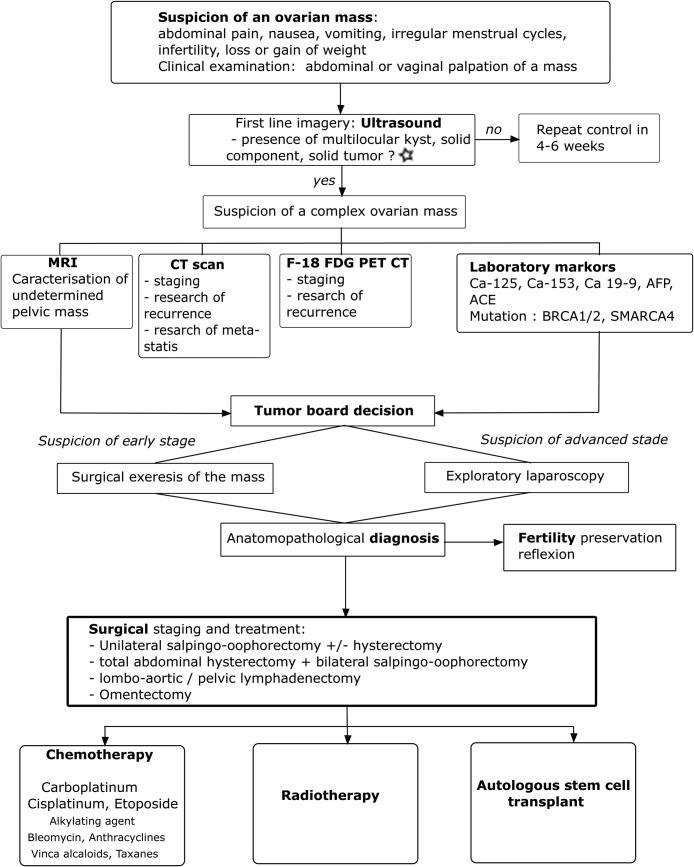
Algorithm 1. Medical management when suspicion of an ovarian SCCOHT: After taking a complete history and a clinical examination, the first line imagery is the vaginal ultrasound. ⋆ If the first ultrasound finds an ovarian volume of more than 20 cm^3^ in premenopausal women and more than 10 cm^3^ in postmenopausal women it is defined as abnormal and the ultrasound should be repeated 4–6 weeks later. Finding of morphological abnormalities (solids areas, papillary projections, complex cystic) needs to be surgically explored as well. If the diagnosis suspicion of SCCOHT persists, complement imagery with MRI, CT scan and PET-CT is needed. Adequate laboratory testing needs to be performed. The surgical decision is taken after discussion with the tumour-board, and the next treatment modalities should be discussed after the anatomopathological confirmation of SCCOHT. The medical management is patient-, age-, morbidity-, disease stage- and fertility preservation desire of the patient- dependent. This algorithm is based on various published reports [5,8,14,16,24,27,29].

We propose this algorithm for medical management when an ovarian mass is suspected, and the diagnosis shows a SCCOHT (Fig. 5).

Research for new treatments includes target therapy with inhibitors of Enhancer of zeste homolog 2 (*EZH2*), a methyltransferase with action on transcription and activation. Alteration of the *SMARCA4* protein, which is highly correlated with SCCOHT [30] and leads to the loss of the BRG1 protein, may increase responsiveness to the *EZH2* inhibitor, tazemetostat [31]. Inhibitors of Programmed cell death protein 1 (PD-1); a protein that down-regulates the immune system and Programmed death-ligand 1 (PD-L1), another protein activator with a role in the immune system, could also be immunologic targets [32].

Mutations of *SMARCA4*, the only recurrently mutated gene in SCCOHT [13], can involve germline or somatic lines, with a high percentage of germline alterations. Concerning this patient, the mutation of *SMARCA4* is a *de novo* sequence variant that follows an autosomal dominant pattern of inheritance. Her first-degree relatives have a 50 % risk of being heterozygous carriers of the same pathogenic variant. Because this mutation is found in about half of the tested patients [5], all patients should be referred to a genetic service. Apart from its diagnostic purpose, genomic profiling results can also have implications in treatment decisions such as target therapy [31,33].

Classified as a miscellaneous tumour [34], SCCOHT shares common genetic and histological characteristics with rhabdoid tumours [35], which are paediatric soft tissue tumours that commonly manifest in the brain or the kidneys [36]. Alterations of *SMARCB1* in rhabdoid tumours or *SMARCA4* in SCCOHT, leads to alterations in the SWItch/Sucrose Non-Fermentable (*SWF/SNF*) a nucleosome remodelling complex [13]. A recent study by Somayyeh et al. showed genomic and epigenomic similarities between SCCOHT and rhabdoid tumours, supporting the hypothesis that SCCOHT should be classified as a rhabdoid tumour (Malignant Rhabdoid Tumour MRT) [13].

SCCOHT affects young and fertile women, giving rise to the question of fertility preservation. Actual guidelines include the use of assisted reproductive techniques to preserve fertility in young female patients with cancer. When surgical care is limited to one ovary, several options exists. Since there is a suspicion of correlation between gonadal activity at the time of chemotherapy administration and the future risk of gonadal failure, ovarian suppression with gonadotropin releasing hormone agonists may be considered for fertility protection during chemotherapy [37]. Ovarian tissue collection or embryo conservation are other assisted reproduction options [38]. So far, there is no link between an increased risk of cancer recurrence and pregnancy [39]. Because of the evolution in oncologic treatments and the improvement of survival rate with SCCOHT, the question of fertility preservation will probably become more relevant.

SCCOHT has a high disease recurrence of about 65 % [1], most often confined to the pelvis, especially in the remaining ovary or the abdomen [2]. We suspected a tumour recurrence in the mass located on the right ovary, with hypercaptation, on the 8-month control CT scan. However, the mass spontaneously disappeared after three months and was possibly due to hormonal stimulation or local inflammation.

This patient declined all treatments after 3 cycles of chemotherapy, even though she was aware of the poor prognosis of this pathology. The medical team attempted to change her mind, including discussion with her parents, but the patient refused to follow any other medical interventions except the imaging control.

It is imperative to remember that a cancer diagnosis can be very traumatic for the patient, and it is the right of the patient not to accept or follow medical advice, especially when the prognosis is poor [40].

## 4 Conclusion

We presented a rare case of a SCCOHT initial stage IV, with complete remission 30 months after diagnosis despite a conservative surgery and only 3 cycles of chemotherapy. The overall survival rate is very low (about 16 %) and patients with extra ovarian manifestations of the disease tend to die within 2 years following diagnosis. Autologous stem cell transplant after high-dose adjuvant chemotherapy seems to lead to the best survival rates. An international collaboration will be needed to standardise practices due of the small number of patients.

## Sources of funding

There was no founding for this case report.

All the authors worked on their free time about this paper.

## Ethical approval

Ethical approval exempt.

## Consent

Written informed consent was obtained from the patient for publication of this case report and accompanying images. A copy of the written consent is available for review by the Editor-in-Chief of this journal on request.

## Author contribution

Marie-Pierre Mathey: carried out thestructure of the article, the literature review and corrected the final version.

Jean Bouquet de La Jolinière: followed the patient, corrected and approved the final version.

Anis Feki: operated the patient, corrected and approved the final version.

*A. Major:* corrected and approved the final version.

*B. Conrad: specialist in genetics, followed the patient.*

*F. Khomsi: operated and followed the patient.*

*D. Betticher: made the oncology follow-up.*

*M. Devouassoux: was the pathologist in charge of the case.*

## Registration of research studies

1 Name of the registry: Research Registry
2 Unique Identifying number or registration ID: 4958
3 Hyperlink to the registration (must be publicly accessible): https://www.researchregistry.com/browse-the-registry

## Guarantor

J. Bouquet de Jolinière MD, PhD.

## Provenance and peer review

Not commissioned, externally peer-reviewed.

## Declaration of Competing Interest

There was no personal or professional interest by writing about this case.

The authors declare any conflict of interest in this article.
